# Effect of Arm Movement and Task Difficulty on Balance Performance in Children, Adolescents, and Young Adults

**DOI:** 10.3389/fnhum.2022.854823

**Published:** 2022-04-25

**Authors:** Thomas Muehlbauer, Mathew W. Hill, Joana Heise, Leander Abel, Ina Schumann, Dennis Brueckner, Simon Schedler

**Affiliations:** ^1^Division of Movement and Training Sciences/Biomechanics of Sport, University of Duisburg-Essen, Essen, Germany; ^2^Center for Sport, Exercise and Life Sciences, School of Life Sciences, Coventry University, Warwickshire, United Kingdom

**Keywords:** postural control, standing, walking, reaching, upper extremities, youth

## Abstract

**Background:**

Studies have shown that restricted compared to free arm movement negatively affects balance performance during balance assessment and this is reinforced when the level of task difficulty (e.g., varying stance/walk conditions, sensory manipulations) is increased. However, it remains unclear whether these findings apply to individuals with differences in the development of the postural control system. Thus, we examined the influence of arm movement and task difficulty on balance performance in children, adolescents, and young adults.

**Methods:**

Static, dynamic, and proactive balance performance were assessed in 40 children (11.5 ± 0.6 years), 30 adolescents (14.0 ± 1.1 years), and 41 young adults (24.7 ± 3.0 years) using the same standardized balance tests [i.e., one-legged stance (OLS) time with eyes opened/closed and/or on firm/foam ground, 3-m beam (width: 6, 4.5, or 3 cm) walking backward step number, Lower Quarter Y-Balance test (YBT-LQ) reach distance] with various difficulty levels under free vs. restricted arm movement conditions.

**Results:**

In all but one test, balance performance was significantly better during free compared to restricted arm movement. Arm by age interactions were only observed for the YBT-LQ and *post hoc* analyses revealed significantly greater performance differences between free and restricted arm movement, especially, in young adults. Arm by age by task difficulty interactions were found for the OLS and the 3-m beam walking backward test. *Post hoc* analyses showed significantly greater performance differences between free and restricted arm movement during high vs. low levels of task difficulty and this was more pronounced in children and adolescents.

**Conclusions:**

Regardless of age, static, dynamic, and proactive balance performance benefited from arm movements and this was especially noted for youth performing difficult balance tasks.

## Introduction

Balance tests (e.g., one-legged stance) are frequently used to quantify balance performance. For the purpose of standardization, the use of arm movement is often limited during test execution (Gribble et al., 2012; Picot et al., 2021). For example, the arms are fixed at the hips or placed flat across the chest. However, it has been shown in several studies (Patel et al., 2014; Hébert-Losier, 2017; Bostrom et al., 2018; Hill et al., 2019; Objero et al., 2019; Wdowski et al., 2021; Sogut et al., 2022) that the restriction of arm movement leads to a deterioration of balance performance (e.g., reduced ability to minimize postural sway), especially in tasks with a high difficulty level. This finding seems to be independent of the subjects’ age, as both youth (Hill et al., 2019; Wdowski et al., 2021) and young adults (Patel et al., 2014; Hébert-Losier, 2017; Bostrom et al., 2018; Objero et al., 2019) showed better balance performance with vs. without the use of arm movement. This is remarkable since young adults have a fully developed postural control system (Woollacott and Shumway-Cook, 1990), thus the use of arm movements should have a relatively small positive impact on balance performance. Contrary in children, the postural control system is not yet fully developed (Hirabayashi and Iwasaki, 1995; Steindl et al., 2006). Therefore, the use of arm movements during testing should have a relatively large positive influence on balance performance, especially when performing tasks with a high difficulty level.

A closer look at the previously reported studies (Patel et al., 2014; Hébert-Losier, 2017; Bostrom et al., 2018; Hill et al., 2019; Objero et al., 2019; Wdowski et al., 2021; Sogut et al., 2022) however reveals that the aforementioned studies only represent indirect comparisons as different methodologies (e.g., standing, walking, mobility, or reaching tasks) were used and only children/adolescents or young adults were investigated. In contrast, a direct comparison between children, adolescents, and young adults in a single study has not been carried out so far. Therefore, it remains unclear whether the positive influence of arm movement on balance performance is independent of age and the applied measurements or whether, due to the age-related development of the postural control system, the promoting influence of arm movement decreases with increasing age.

Thus, the purpose of the present study was to directly compare the effect of free vs. restricted arm movement on balance performance when using tasks of increasing difficulty levels between children, adolescents, and young adults by applying identical measurements to all subjects. Our main hypotheses were that: (1) balance performance would be worse during restricted compared to free arm movement condition, and this effect would be more pronounced for balance tasks with a high difficulty level, and (2) the decrease in balance performance with restricted arm movements would be more pronounced in children and adolescents compared to young adults.

## Material and Methods

### Participants

Forty children, 30 adolescents, and 41 young adults of both sexes participated in this study. The characteristics of the study participants by age group are shown in Table 1. All subjects were healthy and free of any neurological or musculoskeletal impairments. None of the subjects had prior experience with the performed balance tests. Written informed consent and the subject’s assent were obtained from all participants before the start of the study. Additionally, parents’ approval was obtained for minors.

**Table 1:** Characteristics of the study participants (*N* = 111) by age group.

Characteristic	Children (*n* = 40)	Adolescents (*n* = 30)	Young adults (*n* = 41)
Age [years]	11.5 ± 0.6	14.0 ± 1.1	24.7 ± 3.0
Sex [f/m]	22/18	15/15	14/27
Body mass [kg]	44.1 ± 11.8	59.5 ± 14.9	76.1 ± 13.4
Body height [cm]	152.7 ± 7.6	166.7 ± 7.5	177.8 ± 8.9
Body mass index [kg/m^2^]	18.7 ± 3.6	21.2 ± 4.3	23.9 ± 2.7

*Values are means ± standard deviations. F, female; m, male*.

### Testing Procedures

Testing with children and adolescents was carried out during regular physical education lessons at the school gym, while testing with young adults took place during regular practical university courses in a gym hall. Before measurement, all subjects received standardized verbal instructions and a visual demonstration of each test and condition. Afterward, subjects were divided into small groups of five-six subjects and performed the static, dynamic, and proactive balance assessments in a randomized order with each group starting with a different test. Assessment of balance was performed twice (i.e., with and without arm movement) and the testing order was counterbalanced between participants. In the condition without arm movement, the hands were placed on the hips whereas the arms could be moved freely in all directions during the trials with arm movement. The obtained values were noted down on a score sheet for each individual separately. All assessments were performed by experienced raters who were familiar with each test and test circumstances (e.g., noise, brightness) were in accordance with recommendations for balance assessment (Kapteyn et al., 1983).

#### Assessment of Static Balance

**Figure 1:**
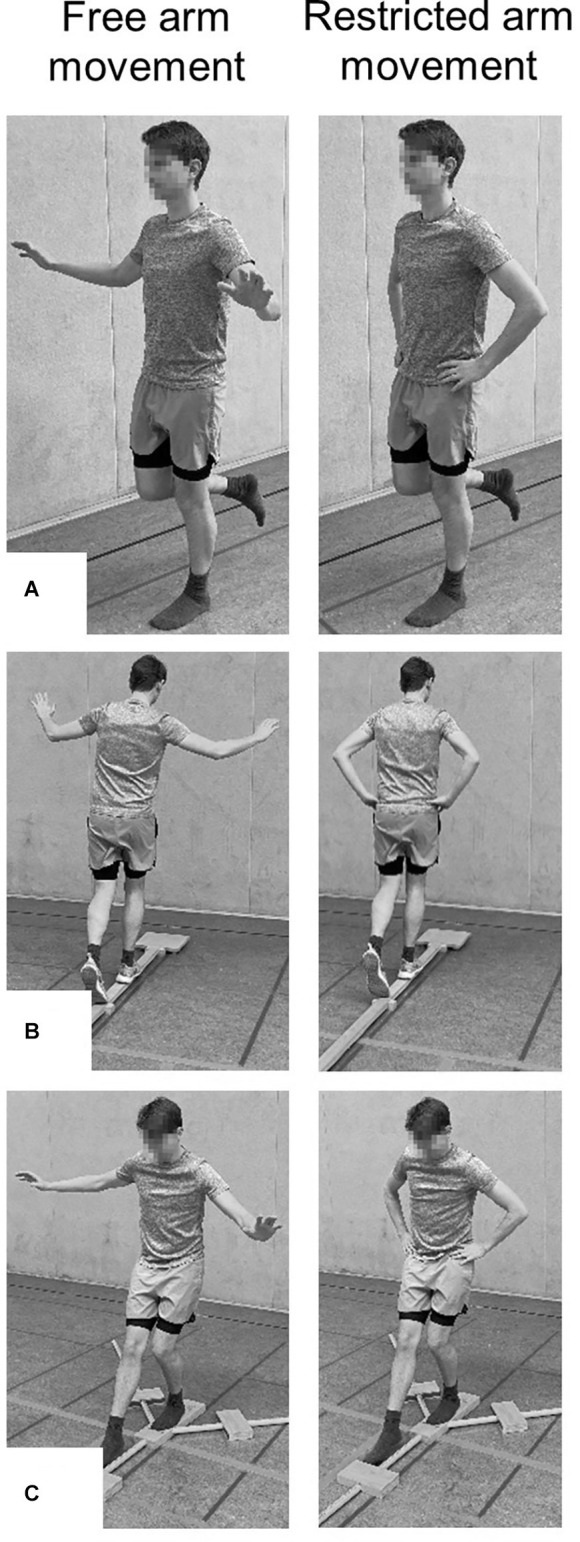
Setup for the assessment of balance using **(A)** the one-legged stance test (i.e., eyes opened, firm ground condition), **(B)** the 3-m beam walking backward test (i.e., beam width: 6 cm), and **(C)** the Lower Quarter Y Balance test (i.e., anterior reach direction).

For the assessment of static balance, the participants were asked to stand without shoes on their dominant leg (i.e., kicking leg as determined per self-report) and gaze fixed on a cross on the nearby wall (Figure 1A). The participants were instructed to perform the one-legged stance (OLS) as long as possible but for a maximum of 60 s (Schilling and Baedke, 1980). The assessment was conducted under four conditions representing different levels of task difficulty in the following order: (1) standing with eyes opened on firm ground (EO, FI); (2) standing with eyes closed on firm ground (EC, FI); (3) standing with eyes opened on foam (i.e., AIREX Balance-pad) ground (EO, FO); (4) standing with eyes closed on foam ground (EC, FO). A trial was classified as invalid if the participants: (1) lost their balance (i.e., stepped with the lifted leg on the ground), (2) opened their eyes during the eyes closed conditions, or (3) unfolded their arms during the restricted arm movement condition. A total of two trials (one familiarization trial followed by one data-collection trial) were executed. The maximal stance time (s) was recorded with a stopwatch to the nearest 0.01 s and was used for further analysis.

#### Assessment of Dynamic Balance

Dynamic balance was assessed using the 3-m beam walking backward test (Figure 1B). The test consisted of three wooden beams that are 3 m long, 5 cm high, and six, 4.5, and 3 cm wide. The participants wore shoes and were instructed to walk backward at a self-selected speed from the beginning to the end of the beam but for a minimum of eight steps (Bös, 2009). A trial ended when the participants: (1) performed eight steps, (2) reached the end of the beam, (3) lost their balance (i.e., stepped on the ground), or (4) unfolded their arms during the restricted arm movement condition. A total of three trials (one familiarization trial followed by two data-collection trials) were executed. The number of steps for both data-collection trials per beam width was noted down on an individual score sheet and was added up resulting in a maximum of 16 steps per beam and used for further analysis.

#### Assessment of Proactive Balance

Proactive balance was assessed by means of the Y-Balance Test Kit (Functional Movement Systems^®^, Chatham, USA). The test kit consisted of a centralized stance platform to which three pipes were attached that represent the anterior (AT), posteromedial (PM), and posterolateral (PL) reach directions. Each pipe is marked in 1.0 cm increments for measurement purposes and was equipped with a moveable reach indicator. Before the Lower Quarter Y-Balance test (YBT-LQ) began, the respective length of the participant’s non-dominant leg was determined in the supine position by measuring the distance from the anterior superior iliac spine to the most distal aspect of the medial malleolus (Plisky et al., 2009). Afterward, participants were asked to reach with the dominant leg as far as possible in the AT, PM, and PL directions while standing with their non-dominant leg on the centralized stance platform (Figure 1C). A trial was classified as invalid if the participants: (1) lost their balance (i.e., stepped with the reach leg on the ground), (2) lifted the stance leg from the stance platform, (3) stepped on top of the reach indicator for support, (4) kicked the reach indicator, or (5) unfolded their arms during the restricted arm movement condition. The maximal reach distance (cm) per reach direction was noted down on a score sheet and was used for further analysis. A total of six trials (three familiarization trials followed by three data-collection trials) were executed. The normalized maximal reach distance [% leg length (LL)] per reach direction was calculated by dividing the absolute maximal reach distance (cm) by LL (cm) and then multiplying by 100. Additionally, the normalized (% LL) composite score (CS) was computed as the sum of the absolute maximal reach distance (cm) per reach direction divided by three times LL (cm) and then multiplied by 100 and used for analysis as well.

### Statistical Analyses

Descriptive data are reported as group mean values and standard deviations after the normal distribution was confirmed using the Shapiro-Wilk test (*p* > 0.05). A arm × age× task difficulty repeated measures analysis of variance (ANOVA) was conducted for static and dynamic balance performance. For measures of proactive balance, a arm × age repeated measures ANOVA was performed. In the case of significant (*p* < 0.05) differences, Bonferroni-adjusted *post hoc* tests (i.e., paired *t*-tests) were performed. Further, effect size ($\eta_{p}^{2}$) was calculated and reported as small (0.02 ≤ $\eta_{p}^{2}$ ≤ 0.12), medium (0.13 ≤ $\eta_{p}^{2}$ ≤ 0.25), and large ($\eta_{p}^{2}$ ≥ 0.26; Cohen, 1988). All statistical analyses were performed using Statistical Package for Social Sciences version 27.0 and the α value was* a priori* set at *p* < 0.05 for all comparisons.

## Results

### Dynamic Balance Performance

**Figure 2:**
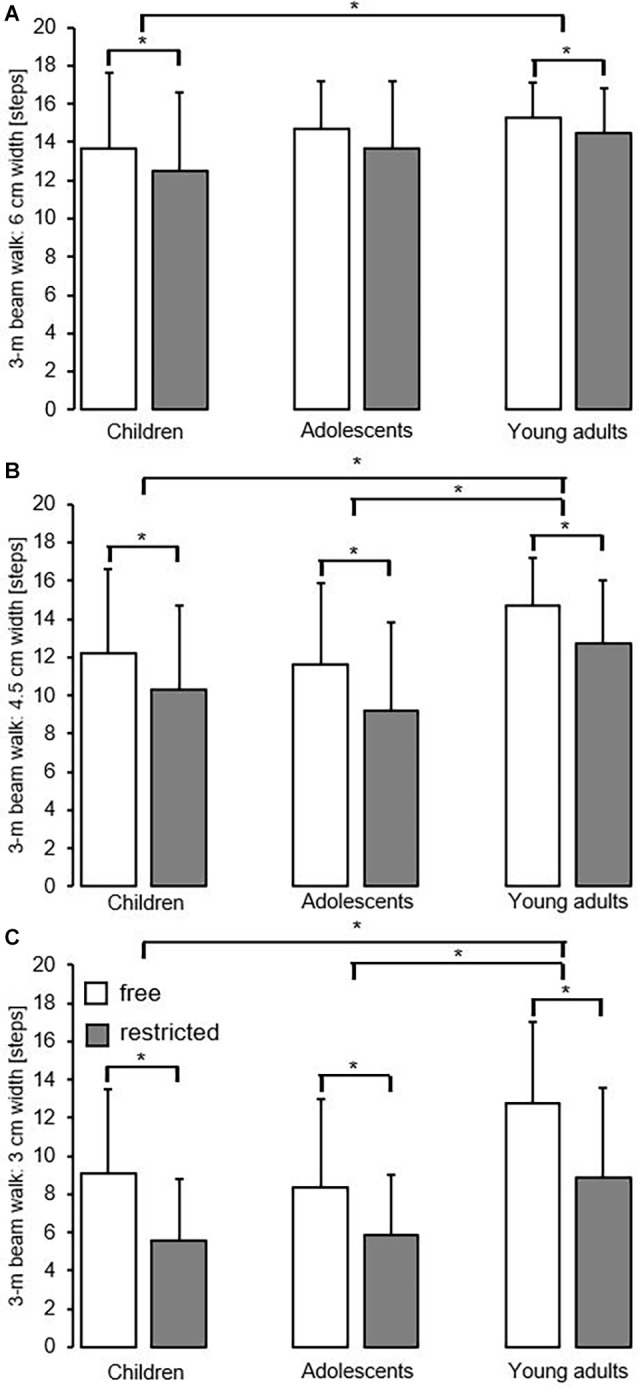
Effect of free vs. restricted arm movement and task difficulty by age group on dynamic balance performance using the 3-m beam walking backward test with a beam width of 6 cm **(A)**, with a beam width of 4.5 cm **(B)**, and with a beam width of 3 cm **(C)**. Values are means ± standard deviations. *Significant difference (*p* < 0.05) between free and restricted arm movement conditions and age groups.

The effect of arm movement and task difficulty by age group on dynamic balance performance is illustrated in Figure 2. Irrespective of beam width, the number of steps during beam walking was significantly lower during restricted compared to free arm movement and larger in young adults compared to adolescents and/or children as indicated by the main effects of arm and age, respectively (Table 2). Furthermore, the ANOVA did not show significant arm × age interactions but yielded a tendency toward a significant arm × age × task difficulty interaction (*p* = 0.067, $\eta_{p}^{2}$ = 0.04). *Post hoc* analyses revealed greater performance differences between free and restricted arm movement during high vs. low levels of task difficulty and this was more pronounced in children (*p* < 0.001) and adolescents (*p* < 0.001) compared to young adults.

**Table 2:** Main and interaction effects of the repeated measures ANOVA per outcome measure.

Test/Outcome	Main effect: arm	Main effect: age	Interaction effect: arm × age	Interaction effect: arm × age × task difficulty
*Static balance*				
OLS time; EO, FI [sec]	0.073 (0.03)	0.003 (0.10)	0.351 (0.02)	<0.001 (0.11)	
OLS time; EC, FI [sec]	0.019 (0.05)	0.001 (0.12)	0.442 (0.02)	
OLS time; EO, FO [sec]	0.002 (0.08)	<0.001 (0.19)	0.642 (0.01)	
OLS time; EC, FO [sec]	<0.001 (0.23)	0.068 (0.05)	0.604 (0.01)
*Dynamic balance*				
6-cm beam walk [steps]	<0.001 (0.11)	0.014 (0.08)	0.888 (0.01)	0.067 (0.04)
4.5-cm beam walk [steps]	<0.001 (0.23)	<0.001 (0.14)	0.847 (0.01)	
3-cm beam walk [steps]	<0.001 (0.35)	<0.001 (0.21)	0.392 (0.02)	
*Proactive balance*				
YBT-LQ: AT reach [% LL]	<0.001 (0.32)	<0.001 (0.16)	0.650 (0.01)	–	
YBT-LQ: PM reach [% LL]	<0.001 (0.52)	0.003 (0.10)	0.155 (0.03)	
YBT-LQ: PL reach [% LL]	<0.001 (0.47)	0.010 (0.08)	0.021 (0.07)	
YBT-LQ: CS [% LL]	<0.001 (0.65)	0.046 (0.06)	0.028 (0.06)	

*Values are *p*-values and effect sizes ($\eta_{p}^{2}$) in brackets. 0.02 ≤ $\eta_{p}^{2}$ ≤ 0.12 indicates small, 0.13 ≤ $\eta_{p}^{2}$ ≤ 0.25 indicates medium, and $\eta_{p}^{2}$ ≥ 0.26 indicates large effects. AT, anterior; CS, composite score; EC, eyes closed; EO, eyes opened; FI, firm ground; FO, foam ground; LL, leg length; OLS, one-legged stance; PL, posterolateral; PM, posteromedial; YBT-LQ, Lower Quarter Y Balance test*.

### Static Balance Performance

**Figure 3:**
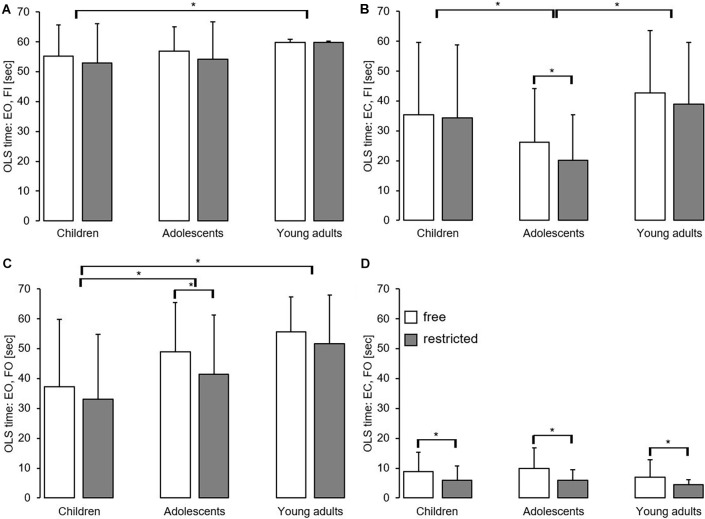
Effect of free vs. restricted arm movement and task difficulty by age group on static balance performance using the one-legged stance with eyes opened on firm ground **(A)**, with eyes closed on firm ground **(B)**, with eyes opened on foam ground **(C)**, and with eyes closed on foam ground **(D)**. Values are means ± standard deviations. *Significant difference (*p* < 0.05) between free and restricted arm movement conditions and age groups. EC, eyes closed; EO, eyes opened; FI, firm ground; FO, foam ground; OLS, one-legged stance.

Figure 3 shows the effect of arm movement and task difficulty by age group on static balance performance. The main effect of the arm indicates that OLS time was significantly lower during restricted compared to free arm movement, except for standing with eyes open on firm ground (Figure 3A). With respect to the main effect of age, our analysis revealed that OLS time was significantly better in young adults compared to adolescents and/or children for all but one stance condition (i.e., EC, FO; Table 2). In addition, the ANOVA failed to detect significant arm × age interactions but showed a significant arm × age× task difficulty interaction (*p* < 0.001, $\eta_{p}^{2}$ = 0.11). *Post hoc* analyses revealed greater performance differences between free and restricted arm movement during high vs. low levels of task difficulty and this was more in pronounced children (*p* = 0.002) and adolescents (*p* = 0.005) compared to young adults.

### Proactive Balance Performance

**Figure 4:**
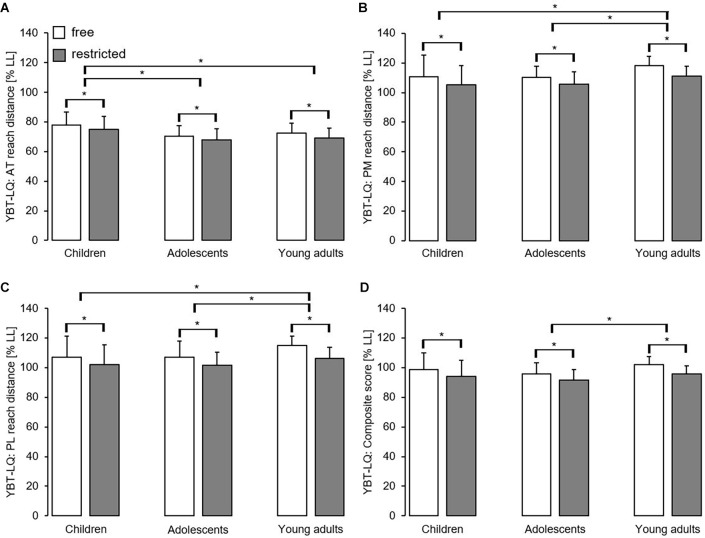
Effect of free vs. restricted arm movement by age group on proactive balance performance using the Lower Quarter Y Balance test consisting of the anterior reach direction **(A)**, the posteromedial reach direction **(B)**, the posterolateral reach direction **(C)**, and the composite score **(D)**. Values are means ± standard deviations. *Significant difference (*p* < 0.05) between free and restricted arm movement conditions and age groups. AT, anterior; LL, leg length; PL, posterolateral; PM, posteromedial; YBT-LQ, Lower Quarter Y Balance test.

Figure 4 displays the effect of arm movement by age group on proactive balance performance. Irrespective of outcome measure, reach distances and the CS were significantly smaller during restricted compared to free arm movement as indicated by the main effect of the arm (Table 2). Further, the main effect of age reached the level of significance in all cases in favor of young adults (except for the AT reach direction). Additionally, the ANOVA yielded significant arm × age interactions for the PL reach direction and the CS. *Post hoc* analyses revealed significantly greater performance differences between free and restricted arm movement in young adults (PL: *p* < 0.001, $\eta_{p}^{2}$ = 0.27; CS: *p* < 0.001, $\eta_{p}^{2}$ = 0.24) compared to children (PL: *p* = 0.001, $\eta_{p}^{2}$ = 0.03; CS: *p* < 0.001, $\eta_{p}^{2}$ = 0.04) and adolescents (PL: *p* < 0.001, $\eta_{p}^{2}$ = 0.06; CS: *p* < 0.001, $\eta_{p}^{2}$ = 0.07).

## Discussion

We directly compared the effect of arm movement and task difficulty on balance performance between children, adolescents, and young adults using identical measurements. Specifically, all subjects performed assessments of static, dynamic, and proactive balance twice, i.e., with (free) and without (restricted) arm movement. For static and dynamic balance testing, the level of task difficulty was increased by manipulating sensory information (i.e., deprivation of the visual input and/or application of a soft stance surface) during standing or by reducing the base of support during walking.

### Effect of Arm Movement on Balance Performance

In accordance with our hypothesis on the positive effect of arm movement on postural control, we found significantly better static, dynamic, and proactive balance performance under free compared to restricted arm movement except for the easiest static balance test condition (i.e., OLS time: EO, FI)which can be attributed to a “ceiling effect” (Hill et al., 2019). These findings correspond with those from earlier studies investigating the effect of arm movement on various measures of balance performance. For instance, Hébert-Losier (2017) investigated young adults (age range: 20–38 years) and reported that YBT-LQ reach distances were significantly greater when the arms moved freely compared to arms akimbo for all three reach directions as well as the CS. Further, Hill et al. (2019) examined children aged 10.6 ± 0.5 years and stated for the free vs. the restricted arm movement condition significantly greater YBT-LQ performances (i.e., all directions and CS) and a faster balance beam walking. Finally, Sogut et al. (2022) recently studied young adults (mean age: 22.7 ± 1.9 years) and again found significantly greater YBT-LQ performances (i.e., PM and PL reach distances and CS) during arms free than arms restricted condition. What are likely reasons for a better postural control during free compared to restricted arm movement? First, arm movements have a positive influence on the whole-body center of mass location. Specifically, corrective movements to counter destabilizing influences can be performed and thus the center of mass can be held better over the base of support (Roos et al., 2008). Second, the moment of inertia increases as more mass is removed from the axis of rotation when the arms are extended (Hill et al., 2019). At the same time, the angular acceleration becomes smaller and more time is gained to perform corrective movements. Third, Newton’s 3rd axiom comes into play, since arm movements enable counter-movements that generate an opposing impulse (Marigold et al., 2003). Fourth, there is a torque due to gravity, which can be compensated by arm movements, thus helping to stabilize the body posture (Patel et al., 2014; Bostrom et al., 2018). The four previously mentioned aspects suggest that the contribution made by arm movements during balance-demanding situations is another postural control strategy in addition to the ankle and hip strategy (Horak and Nashner, 1986).

From the perspective of coaches and therapists, the magnitude of the performance decrease in the YBT-LQ when performed with vs. without arm use is particularly noteworthy. Specifically, the CS dropped from 98.6 to 94.1% LL in children and from 95.9 to 91.7% LL in adolescents, falling to or below the threshold of ≤ 94% LL that Plisky et al. (2006) suggest is associated with an increased risk of lower extremity injury. Further, the direction-specific view showed a performance decrease for the PM (children: from 110.8 to 105.2% LL; adolescent: from 110.2 to 105.8% LL) and the PL reach direction (children: from 106.9 to 102.2% LL; adolescent: from 106.9 to 101.7% LL) below the threshold of an increased injury risk (PM: ≤109% LL; PL: ≤105% LL). Consequently, YBL-LQ assessments in these two age groups (i.e., children and adolescents) should be supplemented by the arms- free condition in order to identify youth at risk as early as possible and to provide appropriate injury preventive treatments.

Further, we detected significant arm by age interactions for measures of proactive but not of static and dynamic balance. Specifically, our *post hoc* analyses revealed significantly greater performance differences between free and restricted arm movement in young adults compared to children and adolescents for the YBT-LQ (i.e., PL reach direction and CS). This finding is contrary to our assumption stating that the effect of arm movement will be lower in adults than in children and adolescents. One possible reason could be that the postural system is fully developed in adults compared to children and adolescents (Shumway-Cook and Woollacott, 1985). In other words, postural control is more automated in adults and less in youth. Consequently, muscle selection, computation, and their sequenced activation are coded on a rather specific level for the former age group and on an unspecific level for the latter age group. Thus, children and adolescents seem to some degree be able to switch between the free vs. restricted arm movement condition during the YBT-LQ execution but this ability seems to be reduced in young adults. Indeed, there is preliminary evidence that postural control becomes more specific with advancing age. For example, Schedler et al. (2021) calculated correlations between types of balance performance where a small coefficient indicates independence from each other (i.e., task-specific) and* vice versa*. They showed that the correlation between dynamic and proactive balance was significantly smaller in young adults (*r* = 0.161) compared to children (*r* = −0.302, *p* = 0.023) and adolescents (*r* = −0.276, *p* = 0.017).

The non-significant arm by age interactions for measures of static and dynamic balance should be considered as part of future more in-depth analyses using kinematic, kinetic, and electromyographic analyses. In sum, our findings indicate that arm movement during the assessment of postural control plays an important role in positively (free arm movement) or negatively (restricted arm movement) modulating balance performance in youth as well as in young adults. From a practitioner’s perspective, the results imply that coaches and therapists are advised to include the use of arm movements in balance training protocols with the goal to progressively change exercise demands from easy (free arm movement) to difficult (restricted arm movement).

### Effect of Task Difficulty on Balance Performance

In line with our hypothesis, we detected that the effect of arm movement restriction was more pronounced for balance tasks with a high than a low difficulty level. That arm contribution increases when the balance task becomes more difficult corresponds with findings from previous studies (Patel et al., 2014; Bostrom et al., 2018; Objero et al., 2019). For example, Bostrom et al. (2018) asked young adults (mean age: 24.3 ± 3.0 years) to walk over three beams of varying widths. They observed that the contribution of upper body movements to dynamic postural control (i.e., torque amplitude/variation) significantly increased when the task difficulty increased, i.e., beam width decreased from 6 cm over 4.5 cm to 3 cm. In addition, Patel et al. (2014) tested young adults (mean age: ~27–28 years) using the tandem stance on a narrow beam. Significantly worse balance performances during fixed vs. outstretched arm movement conditions were observed while standing with eyes closed (more difficult) but not with eyes opened (less difficult). Lastly, Objero et al. (2019) studied young adults (mean age: 20.7 ± 1.3 years) that performed standing tasks of increasing difficulty (i.e., the base of support reduction) with or without arm movement. Balance performances deteriorated significantly more during the restricted arm movement condition for unipedal and tandem stance (more difficult) compared to bipedal stance (less difficult).

Partly in line with our hypothesis, we observed a lower impact of arm movement restriction for balance tasks with a high vs. low difficulty level in adults compared to adolescents and children. Precisely, our *post hoc* analyses of the arm by age by task difficulty interactions revealed that static and dynamic balance performance in young adults was less affected during high vs. low levels of task difficulty when compared to children and adolescents. The greater impact of arm movement restriction on postural control during more vs. less challenging balance tasks in children and adolescents compared to young adults could again be explained by a higher developmental status of the postural control system in young adults compared to youth (Shumway-Cook and Woollacott, 1985). In fact, studies on age differences in balance performance showed that these occurred particularly when sensory manipulations were applied (Hytonen et al., 1993; Steindl et al., 2006). For instance, Steindl et al. (2006) investigated children, adolescents, and young adults using the Sensory Organization Test and found the largest age differences in favor of adults in the particularly difficult condition when both the somatosensory and the visual inputs were manipulated by sway-referencing and eye closing, respectively. Taken together, the present findings indicate that children and adolescents in particular benefit from arm movements during balance assessment when they are asked to perform tasks with a high difficulty level.

The present study has some limitations. With the OLS and the 3-m beam walking backward test, two non-instrumented test devices were used that have an upper limit (i.e., maximal stance time of 60 s; a maximum of 16 steps beam width) that makes it impossible to determine the maximum balance performance. In future studies, instrumented devices, as well as tests without an upper limit of balance performance, should be used to obtain information on maximum balance performance. Further, the ages of the children and adolescents are not much different, so further performance differences may not have emerged. Therefore, future studies should include younger individuals, such as preschoolers, to detect further differences in performance.

## Conclusions

We examined the influence of arm movement and task difficulty on balance performance in children, adolescents, and young adults. Restricted compared to free arm movement lead to worse balance performance, irrespective of age group. Further, the level of task difficulty increased the detrimental effect of restricted arm movement, especially in children and adolescents. If the goal is to quantify maximal balance performance, then arm movements should be allowed particularly in youth performing balance tasks with a high difficulty level. Further, our findings highlight the importance of clearly defining and describing arm position during balance assessment to avoid misinterpretation of balance performance indices and to facilitate experimental replication.

## Data Availability Statement

The raw data supporting the conclusions of this article will be made available by the authors, without undue reservation.

## Ethics Statement

The studies involving human participants were reviewed and approved by Human Ethics Committee at the University of Duisburg-Essen, Faculty of Educational Sciences. Written informed consent to participate in this study was provided by the participants’ legal guardian/next of kin.

## Author Contributions

All authors designed the research question. JH, LA, DB, and IS conducted the testings and data collections. TM and SS analyzed the data. TM, MH, and SS wrote the main parts of the manuscript. All the authors contributed to critical review of draft manuscripts and approved the final manuscript. All authors contributed to the article and approved the submitted version.

## Funding

The support by the Open Access Publication Fund of the University of Duisburg-Essen is acknowledged. The funding body is independent of the design of the study and collection, analysis, and interpretation of data and in writing the manuscript. Open access funding enabled and organized by the project DEAL.

## Conflict of Interest

The authors declare that the research was conducted in the absence of any commercial or financial relationships that could be construed as a potential conflict of interest.

## Publisher’s Note

All claims expressed in this article are solely those of the authors and do not necessarily represent those of their affiliated organizations, or those of the publisher, the editors and the reviewers. Any product that may be evaluated in this article, or claim that may be made by its manufacturer, is not guaranteed or endorsed by the publisher.
